# Prediction of nuclear proteins using nuclear translocation signals proposed by probabilistic latent semantic indexing

**DOI:** 10.1186/1471-2105-13-S17-S13

**Published:** 2012-12-07

**Authors:** Emily Chia-Yu Su, Jia-Ming Chang, Cheng-Wei Cheng, Ting-Yi Sung, Wen-Lian Hsu

**Affiliations:** 1 Graduate Institute of Biomedical Informatics, Taipei Medical University, Taipei, Taiwan; 2 Comparative Bioinformatics, Bioinformatics and Genomics, Centre for Genomic Regulation (CRG), Barcelona, 08003, Spain; 3 Universitat Pompeu Fabra (UPF), Barcelona, 08003, Spain; 4 Bioinformatics Lab., Institute of Information Science, Academia Sinica, Taipei, Taiwan

## Abstract

**Background:**

Identification of subcellular localization in proteins is crucial to elucidate cellular processes and molecular functions in a cell. However, given a tremendous amount of sequence data generated in the post-genomic era, determining protein localization based on biological experiments can be expensive and time-consuming. Therefore, developing prediction systems to analyze uncharacterised proteins efficiently has played an important role in high-throughput protein analyses. In a eukaryotic cell, many essential biological processes take place in the nucleus. Nuclear proteins shuttle between nucleus and cytoplasm based on recognition of nuclear translocation signals, including *nuclear localization signals *(NLSs) and *nuclear export signals *(NESs). Currently, only a few approaches have been developed specifically to predict nuclear localization using sequence features, such as putative NLSs. However, it has been shown that prediction coverage based on the NLSs is very low. In addition, most existing approaches only attained prediction accuracy and Matthew's correlation coefficient (*MCC*) around 54%~70% and 0.250~0.380 on independent test set, respectively. Moreover, no predictor can generate sequence motifs to characterize features of potential NESs, in which biological properties are not well understood from existing experimental studies.

**Results:**

In this study, first we propose PSLNuc (Protein Subcellular Localization prediction for Nucleus) for predicting nuclear localization in proteins. First, for feature representation, a protein is represented by gapped-dipeptides and the feature values are weighted by homology information from a smoothed position-specific scoring matrix. After that, we incorporate probabilistic latent semantic indexing (PLSI) for feature reduction. Finally, the reduced features are used as input for a support vector machine (SVM) classifier. In addition to PSLNuc, we further identify gapped-dipeptide signatures for putative NLSs and NESs to develop a prediction method, PSLNTS (Protein Subcellular Localization prediction using Nuclear Translocation Signals). We apply PLSI to generate gapped-dipeptide signatures from both nuclear and non-nuclear proteins, and propose candidate sequence motifs for putative NLSs and NESs. Then, we incorporate only the proposed gapped-dipeptide signatures in an SVM classifier to mimic biological properties of NLSs and NESs for predicting nuclear localization in PSLNTS.

**Conclusions:**

Experiment results demonstrate that the proposed method shows a significant improvement for nuclear localization prediction. To compare our predictive performance with other approaches, we incorporate two non-redundant benchmark data sets, a training set and an independent test set. Evaluated by five-fold cross-validation on the training set, PSLNuc attains an overall accuracy of 79.7%, which is 4.8% improvement over the state-of-the-art system. In addition, our method also enhances the *MCC *from 0.497 to 0.595. Compared on the independent test set, PSLNuc outperforms other predictors by 3.9%~19.9% on accuracy and 0.077~0.207 on *MCC*. This suggests that, in addition to NLSs, which have been shown important for nuclear proteins, NESs can also be an effective indicator to detect non-nuclear proteins. Most notably, using only a few proposed gapped-dipeptide signatures as input features for the SVM classifier, PSLNTS further enhances the accuracy and *MCC *to 80.9% and 0.618, respectively. Our results demonstrate that gapped-dipeptide signatures can better discriminate nuclear and non-nuclear proteins. Moreover, the proposed gapped-dipeptide signatures can be biologically interpreted and used in further experiment analyses of nuclear translocation signals, including NLSs and NESs.

## Background

### Introduction

In the eukaryotic cells, many essential biological processes take place in the nucleus. Nuclear localization is a complicated set of processes that play a crucial role in the dynamical self-regulation of the cell [1]. To participate in the cell regulation processes, proteins are translocated in and out of nucleus. This import and export are mediated by short binding sites on the protein sequence, called *nuclear localization signals *(NLSs) and *nuclear export signals *(NESs). Both NLSs and NESs have been used as important features to detect nuclear proteins. However, due to a tremendous amount of protein sequences generated from the post-genomic era, NLSs and NESs are not yet well understood from existing experiments by the biologists, and so the set of currently known NLSs and NESs may be incomplete. Therefore, developing computational methods to identify potential NLSs and NESs has become highly desirable to predict nuclear localization.

### Previous works

At present, only a few predictors are designed specifically to identify proteins imported into the nucleus. PredictNLS [2] predicts nuclear proteins based on the presence of known or putative NLSs derived from the contents of NLSdb. NucPred [3] uses regular expression matching and multiple program classifiers induced by genetic programming to detect putative NLSs. NUCLEO [4] incorporates sequence motifs from known NLSs in a support vector machine (SVM) classifier for predicting nuclear localization. NpPred [5] applies SVM classifiers and hidden Markov models (HMM) using *k*-peptide composition and achieves high accuracy based on a data set, in which proteins are filtered at 90% sequence identity [6]. Although general localization prediction methods provide comprehensive information, they do not consider compartment-specific features to optimize for a particular localization site [5]. Besides the above predictors designed to predict nuclear localization proteins, several methods, such as NLStradamus, NetNES, and NoD, have been proposed to detect NLSs and NESs. NLStradamus [7] uses HMMs to predict NLSs in proteins, and NetNES [8] predicts NESs using neural network and HMMs. In addition, NoD [9] applies artificial neural network algorithm to detect nucleolar localization sequences in eukaryotic and viral proteins. Moreover, several methods [10-13] have been developed to further classify nuclear proteins according to their subnuclear localizations. In this study, we will propose a method to improve nuclear localization prediction based on potential NLSs and NESs generated from our analysis of gapped-dipeptide signatures.

### Challenges

Prediction of nuclear proteins presents several challenges. First, methods that integrate biological features only from known or putative NLSs could suffer from the problem of low coverage in high-throughput proteomic analyses due to the lack of information to characterize NESs from nuclear exported proteins. Second, several predictors are implemented on redundant training sets, which might lead to overestimation of the predictive performance. Thus, the performance would be significantly lower if redundant sequences were meticulously removed (e.g., at 25% sequence identity or even less) [4]. Meanwhile, the performance of amino acid composition-based and sequence homology-based methods might be significantly degraded if homologous sequences are not detected [14]. In addition, the *k*-peptide feature representation from amino acid composition-based methods can result in a very large feature dimension during the machine learning procedure, in which an effective feature reduction is highly desirable to reduce dimension. Finally, results of these two types of methods are generally difficult to interpret; therefore, it is difficult to determine which biological features should be used to identify nuclear or non-nuclear proteins and why they work well for prediction. If the features were biologically interpretable, the resultant knowledge could help in designing artificial proteins with the desired properties.

### Our contributions

In this study, we first present a method, PSLNuc (Protein Subcellular Localization prediction for Nucleus), for predicting nuclear localization in proteins. For feature representation, sequence homology information from a smoothed position-specific scoring matrix (PSSM) is incorporated to calculate the weights of gapped-dipeptides. After that, probabilistic latent semantic indexing (PLSI) is used for feature reduction. Finally, the reduced features are applied as input vectors for an SVM classifier. In addition to PSLNuc, we further generate gapped-dipeptide signatures for potential NLSs and NESs, and develop another prediction method, PSLNTS (Protein Subcellular Localization prediction using Nuclear Translocation Signals). To propose candidate sequence patterns of putative NLSs and NESs, we apply PLSI to generate gapped-dipeptide signatures from both nuclear and non-nuclear proteins. Then, we further incorporate only the proposed gapped-dipeptide signatures in an SVM classifier to mimic biological properties of NLSs and NESs in PSLNTS.

Experiment results show that PSLNuc achieves high prediction accuracy, which demonstrates that homology information of gapped-dipeptides reduced by PLSI can significantly enhance the performance. Our analysis suggests that, in addition to NLSs, which have been shown important for nuclear proteins, NESs could also be an effective indicator to detect non-nuclear proteins. Most notably, the overall accuracy of PSLNTS is further improved to 0.809 using only the proposed gapped-dipeptide signatures. This implies that gapped-dipeptide signatures can better discriminate nuclear and non-nuclear localization. In addition, since sequence redundancy tends to overestimate the predictive performance, we incorporate non-redundant data sets and show the general accuracy of nuclear prediction should be approximately 0.800. Finally, since the proposed gapped-dipeptide signatures are biologically interpretable, they can be easily applied to advanced analyses and experimental designs of nuclear translocation signals.

## Results and discussion

### Data sets

To compare the predictive performance with other approaches, we utilize two benchmark data sets of proteins from Swiss-Prot that have been constructed in previous works [2-4]. The training and testing sets are comprised of proteins whose localization sites are experimentally determined. In addition, both the nuclear and non-nuclear proteins are redundancy-reduced using BlastClust with a 10% identity threshold, so that the remaining sequences have no more than 10% identical residues in the aligned regions covering at least 90% of the sequences. Table 1 lists the number of nuclear and non-nuclear proteins in the training and testing data sets. The training and testing sets are available in the supplementary material [see Additional file 1].

**Table 1:** Data sets

	Nuclear	Non-nuclear
Training	2,842	2,606
Testing	564	398

Total	3,406	3,004

Numbers of nuclear and non-nuclear proteins for training and testing.

### Predictive performance on benchmark data sets

Table 2 shows the performance comparison with other approaches based on two benchmark data sets, a training set and an independent test set. First, evaluated by a five-fold cross-validation on the training set, PSLNuc attains an overall accuracy of 0.797, which is a 4.8% (0.048) improvement over the state-of-the-art performance by NUCLEO. In addition, our method also enhances the Matthew's correlation coefficient (*MCC*) from 0.497 to 0.595. Secondly, an independent test data set is incorporated to compare the predictive performance of PSLNuc, NUCLEO, PredictNLS, and NucPred. For the overall accuracy, PSLNuc significantly outperforms the other approaches by 3.9% (0.039) to 19.9% (0.199). Moreover, our method performs better by 0.077 to 0.207 in terms of *MCC*. Experiment results demonstrate that feature reduction by PLSI is able to extract discriminative features for predicting nuclear localization. Meanwhile, our method suggests that proposed smoothed PSSM (smoothPSSM) weighting scheme can better discriminate nuclear and non-nuclear localization by the incorporation of neighboring residues. Finally, in addition to NLSs, which have been shown important for nuclear proteins, NESs could also be an effective indicator to detect non-nuclear proteins.

**Table 2:** Performance comparison

Training Data Set (by five-fold cross-validation)								
	
Method	tp	tn	fp	fn	Sens	Spec	Acc	*MCC*
PSLNuc	2,317	2,030	576	525	**0.815**	**0.779**	**0.797**	**0.595**
NUCLEO	2,157	1,924	682	685	0.759	0.760	0.749	0.497

Independent Test Data Set								

	tp	tn	fp	fn	Sens	Spec	Acc	*MCC*
	
PSLNuc	452	258	140	112	**0.805**	**0.646**	**0.739**	**0.457**
NUCLEO	430	246	152	134	0.760	0.620	0.700	0.380
PredictNLS	153	369	29	411	0.270	0.930	0.540	0.250
NucPred	376	233	165	188	0.670	0.590	0.630	0.250

Performance comparison of different nuclear localization predictors.

### Proposed gapped-dipeptide signatures correspond well with known nuclear translocation signals

To generate gapped-dipeptides for nuclear and non-nuclear localization, we choose ten preferred topics for nuclear and non-nuclear proteins based on localization-preference confidence, respectively. The confidence is calculated by the absolute value of nuclear localization-preference minus non-nuclear localization-preference. For every preferred topic, we select up to twenty most abundant gapped-dipeptides. Finally, the resultant gapped-dipeptide signatures for nuclear and non-nuclear proteins are listed in Table 3 and Table 4, respectively. We compare the generated gapped-dipeptide signatures with known experimentally determined NLSs and NESs in the biological literature and databases. The signatures that have been reported as motifs for NLSs and NESs are shown in bold face in Table 3 and Table 4, respectively. It is observed that many gapped-dipeptide signatures are motifs critical for predicting nuclear localization, especially for NLSs. Our experiment results show that the proposed method can capture biological features of nuclear and non-nuclear proteins.

**Table 3:** Gapped-dipeptide signatures for nuclear proteins

Gapped-dipeptide signatures for nuclear proteins									
G2P	G11P	G8P	P11P	P5P	P3Q	P5Q	**K1K**	**K6K**	**K10K**
**K2K**	**K5K**	**K0K**	**K3K**	**K4K**	E4E	E1E	E8E	E0E	P1T
P0T	P6T	P10T	**G2G**	**G1G**	**G5G**	P1S	S6P	P3G	G4P
P0G	Q11Q	Q10Q	Q5Q	**R13R**	**R11R**	**R10R**	**R9R**	**R12R**	**R7R**
**R3R**	**R4R**	C8S	S2C	S6C	N6P	P12N	S9D	E12D	D12D
D4D	D13D	S12T	S8T	S10T	**K0R**	**K2R**	**R2R**	**R0R**	**R0K**
**R2K**	**K1R**	**R1K**	**R3K**	**K4R**	M3P	P12M	**R1H**	Q8R	Q2H
H0R	S7Q	Q12S	S13H	S10H	S2H	H6S	S12H	P7Y	P4Y
Y5P	N3N	N6N	N0N	**K1H**	**H0K**	**H6K**	**K10H**	D2S	D5S
K8S	D0S	N8N	H8N	H5N	**S6L**	T4E	E13S	T6E	**S13S**
**S10S**	**H0H**	**H4H**	**H1H**	**H13H**	N0E	N0K	N4K	K1N	D2T
G6L	G8L	E8C	K1C	K5C	K7C	C2C	C0C	K3C	C4K
H8C	H12C	H13C	H10C	D6I	D10I	D12I	A13Q	A6Q	Q9A
Q6A	**S11S**	M3S	Q13T	K9T	Q1T	Q9T	N1S	S7N	N8S
H1I	I0H	E12I	S2E	E11I	D6A	S3D	D8A	M13Q	M3Q
L13S	L12S	V6S	S9N	**S13G**	E9S	**S3S**	N0S	S3N	H1Q
H10Q	H3Q	H5Q	T7E	T8E	T12E	T9E	W13K	W11K	N11I
I12N	M13D	M2D	F13D	L1H	H1L	A3H	L5H	**R0G**	A9H
H10A	**P3R**	A10H							

Our method propose 183 gapped-dipeptide signatures for nuclear proteins. Signatures that have been reported as motifs for nuclear localization signals (NLSs) are shown in bold face.

**Table 4:** Gapped-dipeptide signatures for non-nuclear proteins

Gapped-dipeptide signatures for non-nuclear proteins									
**L2L**	**L2F**	**F5L**	**F2L**	**M0V**	**M1V**	**V0M**	**V1M**	**V3V**	**V2V**
A3V	A8V	A12I	A6I	A4I	I2A	E11L	L11Q	Q11L	L11E
Q5W	Q13W	S7W	Q12W	I13I	**I9I**	I12I	I10I	Y6Y	Y0Y
Y5Y	Y12Y	V0N	L2N	I2Q	I2S	R11A	R8A	R1A	K1L
L1K	L0K	F10A	F11A	F11S	F10S	L8A	L4A	L5A	A6L
F3F	F4F	F10F	F13F	**L8L**	**L3L**	**I3L**	**L12L**	V11D	V13D
V7D	V4D	I5E	I4D	I12E	E5I	W8P	W9P	W10P	W3P
V5V	V13V	G2V	G11V	S2S	S4S	A5S	S9S	W1H	H10W
W8H	Y10W	M3M	M2M	M13M	M0M	T0T	T13T	T7T	T1T
H1Y	H3Y	H13Y	H7Y	F12C	F9C	F0C	F10C	A7K	A3K
A10A	A4A	A5A	A9A	Y2G	Y0G	G0Y	G0F	F2K	K0F
K3F	Q13L	L13Q	L1T	L10Q	C12I	I12C	C10I	C3I	W4E
W0L	W5L	W1L	Y7T	Y7V	Y6V	V5Y	E1M	L9K	R11M
I10K	I13K	F13K	T1K	K5I	K2V	H10L	L12H	H8L	E13L
E13I	E10V	N13L	K13N	K12N	I9N	V10N	V12N	V1N	R10Q
D3R	R2D	V7G	M11G	V13G	L3G	I6N	I5N	V9I	I11N
K7L	V4H	H12V	V8H	H4V	F9E	D13L	D12L	L3D	D9L
H11M	L10M	M7H	H12I	E9L	E11A	E12A	A5E	E10A	I4N
N0I	N13I	N9I							

Our method propose 183 gapped-dipeptide signatures for non-nuclear proteins. Signatures that have been reported as motifs for nuclear export signals (NESs) are shown in bold face.

**Figure 1:**
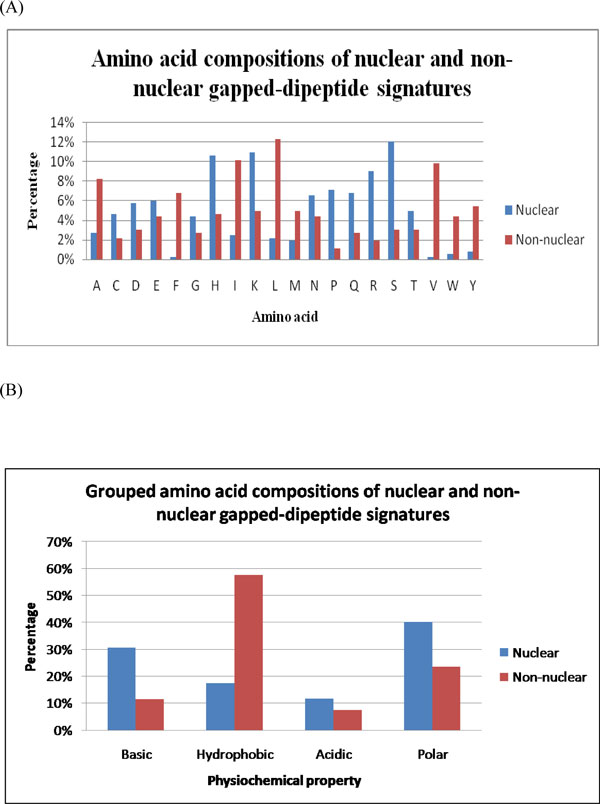
**Physicochemical analyses of gapped-dipeptide signatures**. (A) Amino acid compositions and (B) grouped amino acid compositions of gapped-dipeptide signatures for nuclear and non-nuclear proteins.

Figure 1(A) and 1(B) illustrate further analyses of amino acid composition and grouped amino acid composition of selected gapped-dipeptide signatures, respectively. When analyzing physicochemical properties in grouped amino acid composition, each amino acid is grouped into one of the four categories: aromatic (FYW), charged (DEHKR), nonpolar (AIGLMV), and polar (CNPQST). From Figure 1(A), it is observed that nuclear signatures show great preferences for Arginine (R), Histidine (H), and Lysine (K), which can be discovered in most NLSs; while non-nuclear signatures exhibit high compositions in Leucine (L), Isoleucine (I), Phenylalanine (F), Valine (V), Methionine (M), and Proline (P), which occur frequently in NESs. In addition, as shown in Figure 1(B), nuclear signatures prefer basic (HKR) or polar (CGNQSTY) amino acids, and non-nuclear ones favor hydrophobic (AFILMPVW) amino acids. Experiment results demonstrate that the amino acid preferences of selected nuclear and non-nuclear signatures correspond well with biological knowledge [15].

### Gapped-dipeptide signatures better discriminate nuclear and non-nuclear proteins

To demonstrate that our method can capture biological properties of putative NLSs and NESs, we further examine the predictive performance using only the proposed gapped-dipeptide signatures. In PSLNTS, we incorporate 366 selected gapped-dipeptide signatures in an SVM classifier to see whether gapped-dipeptide signatures can better discriminate nuclear and non-nuclear proteins. Table 5 compares the performance of PSLNTS, PSLNuc, and NUCLEO. Using only the 366 gapped-dipeptide signatures, PSLNTS performs slightly better than PSLNuc by nearly 0.010 (1%) and 0.023 for overall accuracy and *MCC*, respectively. Experiment results demonstrate that the selected signatures can capture biological properties of NLSs and NESs, and thus, can resolve the ambiguity to discriminate nuclear and non-nuclear proteins.

**Table 5:** Performance comparison using gapped-dipeptide signatures

	Training Set (by five-fold cross-validation)							
	
	tp	tn	fp	fn	Sens	Spec	Acc	*MCC*
PSLNTS	2,429	1,978	628	413	**0.855**	**0.759**	**0.809**	**0.618**
PSLNuc	2,413	1,964	642	429	0.815	0.779	0.797	0.595
NUCLEO	2,157	1,924	682	685	0.759	0.760	0.749	0.497

Performance comparison of PSLNuc, PSLNTS, and NUCLEO.

## Conclusions

In this study, we first incorporate gapped-dipeptides weighted by a smoothPSSM encoding and reduced by PLSI to predict nuclear localization in PSLNuc. Our results show that PSLNuc significantly improves the predictive performance compared to the state-of-the-art system. Experiment results also suggest that, in addition to NLSs, which have been shown important for nuclear proteins, NESs can also be an effective indicator to detect non-nuclear proteins. Secondly, we apply only a few proposed gapped-dipeptide signatures in PSLNTS and further enhance the accuracy and *MCC *to 0.809 and 0.618, respectively. This demonstrates that gapped-dipeptide signatures can better discriminate nuclear and non-nuclear localization. Most notably, the proposed gapped-dipeptide signatures could be biologically interpreted and used in further experiment studies of nuclear translocation sequences, including NLSs and NESs.

## Methods

In this study, we extend our previously proposed method for general localization site prediction [16] and formulate the nuclear protein prediction as a document classification problem. In our previous work, we incorporated gapped-dipeptides as feature representation and PLSI as feature reduction for predicting protein localization sites. The method was inspired by word representation and word vector reduction in the research of document classification, where a document is assigned to one or several categories according to its content. Similarly, prediction of nuclear localization can be formulated as a document classification problem, in which a protein sequence is regarded as a document's content and its subcellular localization classes can be treated as categories of the document. Classification of documents is often solved in steps described as follows. First, for feature representation, each document is represented by a feature vector, where each word denotes a feature and its feature value represents the weight of a word in the document. Second, due to a high-dimensional feature space of words in a document, features are further reduced to enhance prediction accuracy and prevent overestimation [17]. Finally, reduced features are incorporated as input vectors in machine learning approaches for predicting document categories. To calculate the weights of each word, a standard PSSM encoding was incorporated to predict general protein localization in our previous study [16]. However, it has been shown that a smoothPSSM encoding scheme is more effective to predict protein structure and function [18]. In this study, we incorporate a new smoothPSSM encoding scheme and extend our approach to predict nuclear localization for bioinformatics analysis. The task is a large-scale analysis of nuclear proteins, including prediction of nuclear localization and analyses of nuclear translocation signals. To solve these problems, we propose a prediction method in which proteins are represented by gapped-dipeptides from smoothPSSM and PLSI is incorporated for feature reduction. Next, the feature representation, feature weighting, feature reduction, system architecture, and evaluation measures are described in the following sections.

### Feature representation - gapped-dipeptides as words of proteins

When proteins are considered as documents, several types of word representation have been used, such as amino acid compositions [19] and *k*-peptide compositions [20]. Specifically, a dipeptide (*k *= 2) composition can be considered as a bi-gram word representation. However, peptides with gaps cannot be represented by a *k*-peptide composition. In addition, feature vectors with high dimensions could be generated if *k*-peptide compositions are used to represent remote sequence information. For instance, the dimension of a feature vector reaches up to 8,000 if a tri-peptide composition is considered. To distinguish nuclear and non-nuclear proteins, we incorporate gapped-dipeptide representation used in our previous work [16] to represent sequence features in proteins. A gapped-dipeptide *AdB *represents the sequence patterns that two amino acids *A *and *B *are divided by *d *residues. When we consider gapped-dipeptides up to *u *gapped distance, the feature dimension of gapped-dipeptides in a protein is the total number of probable combinations, that is 20 × 20 × (*u*+1). For instance, when *u *= 16, a feature vector of 6,400 (= 20 × 20 × 16) dimension is used to represent a protein sequence. Due to computational and time complexity, tri-peptides or other *k*-peptides are not considered in this study.

### Feature weighting - homology information from position-specific scoring matrix

#### Standard position-specific scoring matrix

**Figure 2:**
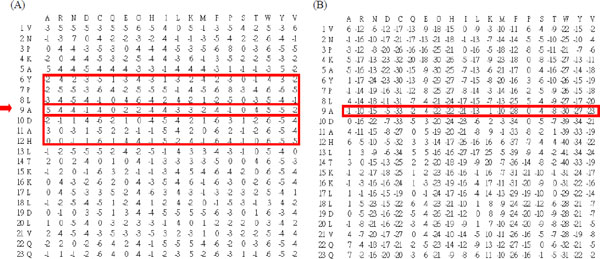
**Construction of a smoothed PSSM profile**. Transformation of (A) a standard PSSM profile into (B) a smoothed PSSM profile using *w *as 7.

As shown in Figure 2(A), a standard PSSM in a protein *P *of *n *amino acids is denoted as an *n *× 20 matrix, where *n *denotes the residues in protein *P *and 20 represents the twenty amino acids. The elements in the standard PSSM denote the amino acid substitution log-likelihood of different residues in the protein *P *[21]. We incorporate PSI-BLAST to generate homology information from PSSM. The parameters in PSI-BLAST are set to *j *= 3 (three iterations), *e *= 10^-3^, and the searched database is NCBI nr.

#### Smoothed position-specific scoring matrix

In this study, we incorporate a smoothPSSM encoding scheme, which has been shown effective for protein-RNA binding site prediction by considering the dependency or correlation among neighboring residues [18]. In a standard PSSM profile, the log-likelihood at each position is calculated based on an assumption that each position is independent from the others. Inspired by the consideration of adjacent pixels used in the spatial domain method from the research field of image processing, a new encoding scheme is proposed to consider the dependency among surrounding neighbors. We use a sliding window of size *w *to incorporate the homology information from upstream and downstream residues. In the construction of a smoothPSSM, each row vector of a residue *α_i _*is represented and smoothed by the summation of *w *surrounding row vectors (*V_smoothed_i _*= *V_i-(w-1)/2 _*+ ... + *V_i _*+ ... + *V_i+(w-1)/2_*). For the N-terminal and C-terminal of a protein, *(w-1)/2 *zero vectors are appended to the hand or tail of a smoothPSSM profile. Using the smoothPSSM encoding scheme, the feature vector of a residue *α_i _*is represented by (*V_smoothed_i-(w-1)/2_*, ..., *V_smoothed_i_*, ..., *V_smoothed_i+(w-1)/2_*). Here, we adopt gapped-distance as 13 and smoothing window sizes as 7 according to our previous studies [16,18]. Figure 2(B) illustrates an example of a smoothPSSM profile. At position 9, the corresponding value of amino acid 'A' represented by a smoothPSSM encoding is the sum of [(-2) + (-2) + (-3) + 5 + (-2) + 3 + 0]. The log-likelihoods in a smoothPSSM are normalized to 0~1 based on a logistic function [22]:

(1) $$f\left(x\right)=\frac{1}{1+e^{-x}},$$

where *x *is a log-likelihood in a smoothPSSM.

#### TFsmoothPSSM weighting scheme

Using smoothPSSM, a TFsmoothPSSM encoding is calculated in the following steps. For a protein sequence *P *of length *n*, a gapped-dipeptide A*d*B of *P *contains smoothPSSM elements that corresponds to sequence pattern *P_i_dP_i+d+1 _*for 1≦*i*≦*n*-(*d*+1), in which *P_i _*represents the *i*th amino acid of *P*. Take a protein sequence MKGIKSKMLS as an example. The gapped-dipeptide M2I only shows one occurrence in the protein. However, the weighting of M2I can be contributed from different gapped-dipeptides of the protein in a smoothPSSM (i.e., M2I, K2K, G2S, I2K, K2M, S2L, and K2S). The word weighting of a gapped-dipeptide A*d*B in *P *is calculated by

(2) $$W\left(AdB,P\right)=\underset{1\leq i\leq n-\left(d+1\right)}{\sum }sf\left(i,A\right)\times sf\left(i+d+1,B\right)$$

in which *sf*(*i, A*) represents a normalized log-likelihood in the smoothPSSM element of the *i*th row and the *A*th column. From the sample sequence, the weighting of M2I using a smoothPSSM profile is calculated as *sf*(1, M) × *sf*(4, I) + *sf*(2, M) × *sf*(5, I) + ... + *sf*(7, M) × *sf*(10, I). A protein is denoted as a feature vector consisting of gapped-dipeptides, where each gapped-dipeptide is weighted by TFsmoothPSSM encoding scheme. Finally, the feature vector is normalized to a range of 0 to 1.

### Feature reduction - probabilistic latent semantic indexing

#### Probabilistic latent semantic indexing

Hofmann proposed PLSI based on an aspect model for feature reduction [17]. PLSI aims at identifying and discriminating between different contexts of word usage without the help from a thesaurus or dictionary. It can identify semantic similarities by classifying together words with identical or similar meanings. Latent topic variables t ∈ *T *= {*t*_1_, ..., *t_K_*} for co-occurrence data are incorporated to associate each observation in the aspect model. We use a latent topic variable *t *to model the weight of a word *w *in a document *d*, which is regarded as a joint probability *P*(*w, d*) between *w *and *d*. Therefore, *P*(*w, d*) is calculated by

(3) $$P\left(w,d\right)=P\left(d\right)P\left(w|d\right),P\left(w|d\right)=\underset{t\in T}{\sum }P\left(w|t\right)P\left(t|d\right)$$

in which *P*(*w*|*t*) represents the conditional probability of a word *w *conditioned on a topic *t*, and *P*(*t*|*d*) represents the weighting of a topic variable *t *in a document *d*. It is assumed that the word distribution given a topic class is conditionally independent of the document *d*, i.e., *P*(*w*|*t, d*) = *P*(*w*|*t*). Therefore, the original feature dimension |*W*| of the word vector is greatly reduced to the number of latent topic variables |*T*|.

#### Probabilistic latent semantic indexing training and testing

We incorporate the same procedure as described in our previous work [16] to train and test PLSI model. First, for PLSI training, the parameters *P*(*w*|*t*) and *P*(*t*|*d*) are fitted by an iterative expectation-maximization algorithm, in which *P*(*t*|*d*) is estimated in the expectation (E) step and *P*(*w*|*t*) is recalculated in the maximization (M) step. Then, after training, the calculated probability of a word conditioned on a topic *P*(*w*|*t*) is used to estimate the *P*(*t*|*d'*) for new documents *d' *through a folding-in process [17] in PLSI testing.

#### Feature reduction by probabilistic latent semantic indexing

The application of PLSI can not only reduce feature dimension, but also extract semantic relationships of gapped-dipeptides. During PLSI feature reduction process, gapped-dipeptides with similar meanings or preferences are grouped together in a semantic topic, and then the topic preferences to nuclear or non-nuclear localization can be identified. If we can select an appropriate topic size in feature reduction, the mappings of feature vectors from the gapped-dipeptide space to latent semantic topic space can greatly increase the learning efficiency and performance. One way to approximate the reduced feature size is based on latent semantic indexing (LSI). First, singular values of LSI are calculated and sorted in a decreasing order. After that, we select *t *as the reduced feature size of LSI if the *t*-th largest singular value is close to zero. Although PLSI is not identical to LSI, the number of singular values larger than zero is reasonably estimated by the number of the PLSI reduced dimensions. We take the reduced number of topics as 80 according to our previous study [16].

### System architecture

**Figure 3:**
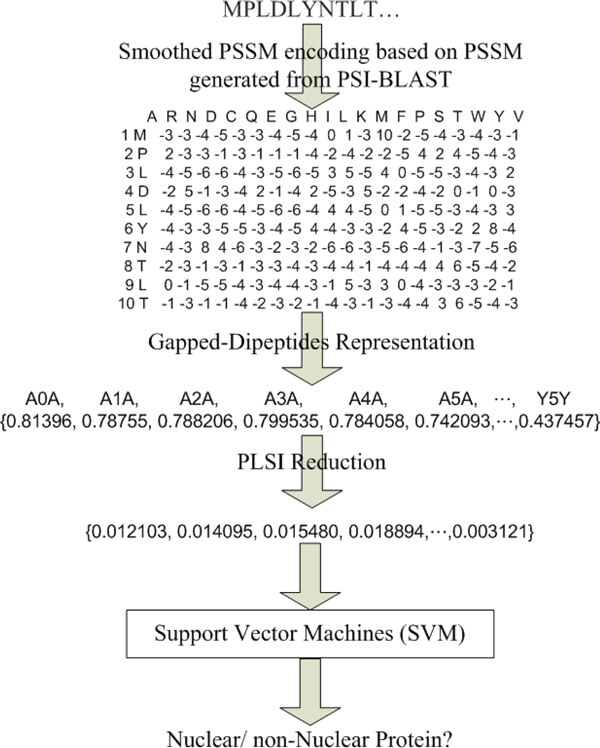
**System architecture of PSLNuc**. System architecture of PSLNuc based on support vector machines using reduced/transformed feature vectors.

Prediction of protein nuclear localization can be regarded as a two-class classification problem. For a two-class classification problem, the SVM classifier has been demonstrated effective in classification [23]. Our prediction method consists of a one-versus-one SVM classifier corresponding to nuclear and non-nuclear proteins. For the SVM classifier in our system, we incorporate libsvm [23], where probability estimation values are shown to determine the classification confidences [24]. In libsvm, we use the Radial Basis Function (RBF) kernel and tune cost (*c*) and gamma (*γ*) parameters based on five-fold cross-validation. Here we propose PSLNuc for predicting nuclear proteins using smoothPSSM and PLSI. Our method applies gapped-dipeptides weighted by TFsmoothPSSM scheme to represent features of a protein. After that, the feature vectors are reduced by PLSI and incorporated as input vectors for an SVM classifier. The system architecture of PSLNuc is illustrated in Figure 3. For each protein, PSLNuc predicts its localization according to these steps:

1. Perform PSI-BLAST to calculate a standard PSSM for the protein.

2. Construct a smoothPSSM profile based on the standard PSSM.

3. Use gapped-dipeptides to represent the protein and incorporate TFsmoothPSSM encoding scheme to determine the weights in a feature vector.

4. Apply PLSI to reduce the feature vector.

5. Use the reduced feature vector as input and run the one-versus-one (nuclear and non-nuclear) SVM classifier.

### Nuclear protein prediction based on proposed translocation signals

For localization-topic preference identification, we divide the training data sets into nuclear and non-nuclear proteins to examine preferred topics. The localization-topic preference of a topic is computed as the average of topic weights from the proteins in a localization site. A topic is identified as showing preference to a localization site if its localization-topic preference is larger than the other site. For nuclear localization prediction, we divide the proteins into two categories, and select 10 top preferred topics for nuclear and non-nuclear proteins according to localization-topic preference, respectively. To list gapped-dipeptides of interest, for each topic, up to 20 (depending on the number gapped-dipeptides in the topic) most frequent gapped-dipeptides are selected. After that, we incorporate only the proposed gapped-dipeptide signatures for nuclear and non-nuclear proteins for predicting nuclear localization. We apply the proposed gapped-dipeptide signatures to capture biological properties and mimic translocation mechanisms of nuclear translocation.

### Evaluation measures

For predictive performance comparison, the same evaluation measures applied in other approaches [2-5] are incorporated. Evaluation measures include sensitivity (*Sens*), specificity (*Spec*), accuracy (*Acc*; also known as success rate), and *MCC *defined in Equation (4), (5), (6), and (7) below:

(4) $$Sensitivity=\frac{TP}{TP+FN}$$

(5) $$Specificity=\frac{TN}{TN+FP}$$

(6) $$Accuracy=\left(TP+TN\right)/)N$$

(7) $$MCC=\frac{TP\times TN-FP\times FN}{\sqrt{\left(TP+FN\right)\left(TP+FP\right)\left(TN+FP\right)\left(TN+FN\right)}}\;$$

where *TP, TN, FP, FN*, and *N *represent the number of true positives, true negatives, false positives, false negatives, and total number of protein sequences, respectively. For an objective comparison with other approaches that use five-fold cross-validation, we also apply five-fold cross-validation to evaluate our predictive performance.

## Competing interests

The authors declare that they have no competing interests.

## Authors' contributions

ECYS developed the method, carried out the computational predictions, and drafted the manuscript. JMC and CWC participated in the experimental design and supplied additional insights regarding the analyses. TYS and WLH refined the manuscript. All authors read and approved the final manuscript.

## Supplementary Material

Additional file 1 **Data sets**. The protein sequences in the benchmark data sets used for training and testing are listed as supplementary data. Click here for file
